# The influence of anticipated pride and guilt on pro-environmental decision making

**DOI:** 10.1371/journal.pone.0188781

**Published:** 2017-11-30

**Authors:** Claudia R. Schneider, Lisa Zaval, Elke U. Weber, Ezra M. Markowitz

**Affiliations:** 1 Department of Psychology, Columbia University, New York, New York, United States of America; 2 Center for Research on Environmental Decisions, Columbia University, New York, New York, United States of America; 3 Center for Decision Sciences, Columbia University, New York, New York, United States of America; 4 Department of Psychology, Princeton University, Princeton, New Jersey, United States of America; 5 Woodrow Wilson School of Public and International Affairs, Princeton University, Princeton, New Jersey, United States of America; 6 Andlinger Center for Energy and the Environment, Princeton University, Princeton, New Jersey, United States of America; 7 Department of Environmental Conservation, University of Massachusetts Amherst, Amherst, Massachusetts, United States of America; University of Melbourne, AUSTRALIA

## Abstract

The present research explores the relationship between anticipated emotions and pro-environmental decision making comparing two differently valenced emotions: anticipated pride and guilt. In an experimental design, we examined the causal effects of anticipated pride versus guilt on pro-environmental decision making and behavioral intentions by making anticipated emotions (i.e. pride and guilt) salient just prior to asking participants to make a series of environmental decisions. We find evidence that anticipating one’s positive future emotional state from green action just prior to making an environmental decision leads to higher pro-environmental behavioral intentions compared to anticipating one’s negative emotional state from inaction. This finding suggests a rethinking in the domain of environmental and climate change messaging, which has traditionally favored inducing negative emotions such as guilt to promote pro-environmental action. Furthermore, exploratory results comparing anticipated pride and guilt inductions to baseline behavior point toward a reactance eliciting effect of anticipated guilt.

## Introduction

A growing body of research points to the central role that the anticipation of future affective states plays in shaping future- and other-oriented decision making [1, 2]. Emotionally engaging pro-social behaviors, such as helping victims in need or donating money to a charitable organization, may be particularly sensitive to such processes [3]. The anticipation of future affective states, both positive and negative, may be a powerful motivator of taking positive actions on behalf of others, particularly among those that carry strong personal and/or cultural norms of caring for others. In the present study, we extend the growing literature on anticipated emotions to examine how two specific states–anticipating feeling pride and guilt—compare in their influence on pro-environmental decision making and behavioral intentions.

### Anticipated emotions and decision making

Mellers and McGraw [4] provide a framework for understanding how decision makers’ anticipation of the future affective consequences of actions yet-to-be-taken influence decisions made in the present. Such cognitive predictions about future emotions are referred to as ‘anticipated emotions’ [5]. In simple terms, people tend to avoid taking actions that could result in negative emotions (e.g., guilt, sadness) and to pursue those that will result in positive states (e.g., pride, joy).

An emerging body of work suggests that the anticipation of future emotional states may play a particularly powerful role in shaping pro-social and other-oriented behaviors [2, 6]. For example, when making the decision of whether or not to donate bone marrow, contemplating future feelings of guilt related to inaction can lead to donating [7]. Similarly, when playing a bargaining game with others, anticipating feelings of pride about fair gameplay has been shown to support fair behavior [6]. These and related results suggest that encouraging individuals to anticipate how they will feel about upcoming other-oriented decisions may be a novel and under-appreciated pathway to promoting pro-social behaviors. Towards this end, the current paper explores and compares whether the anticipation of specific future emotional states can influence pro-environmental decision making.

### Pro-environmental behavior and the anticipation of pride and guilt

Recent research suggests links between anticipated emotions and sustainable behavior, focusing on two discrete types of emotions in the environmental domain: feelings of guilt and pride. These emotions are highly relevant to pro-environmental motivations, as both pride and guilt orient individuals to social concerns [8, 9] and moral considerations [10–12]. Moreover, both emotions are frequently targeted by environmental campaigners and communicators. This is particularly true of guilt, which is assumed by many environmentalists to be a powerful motivator of reparative [1, 13, 14] and preventative action [15, 16]. Although guilt-oriented approaches to encouraging pro-environmental behavior can be successful [15, 16, 14], it is important to note that they also run the risk of alienating people and ultimately inhibiting sustainability [17–19]. A smaller number of campaigns have attempted to link environmental conservation with positive emotions (e.g., www.rare.org). Although relatively few controlled demonstrations exist, some research suggests that pride-oriented approaches to behavior change could be quite successful and robust [20–22].

If the primary emotional reaction people experience when exposed to environmental degradation is negative, then pro-environmental responses may be aimed at relieving that negative feeling. Supporting this notion, a number of studies have shown a positive relationship between guilt and pro-environmental action [16, 14]. Elgaaied [15], for example, demonstrated a relationship between anticipated guilt and pro-environmental behavior linked to recycling patterns in France. Carrus, Passafaro and Bonnes [23] investigated public transportation use and recycling behavior and similarly found a positive effect of negative anticipated emotions on the desire to act pro-environmentally. Lu and Schuldt [14] explored the relationship between incidental guilt and support for climate change policy and report a positive main effect of a guilt induction compared with no emotional induction.

On the other hand, preventing environmental degradation may entail positive feelings; thus, a desire to experience positive emotions may also motivate pro-environmental action. Although benefits of associating positive emotional experiences with pro-environmental actions have been suggested [23, 24], little empirical work has examined whether such a correlational or causal connection in fact exists. Two recent exceptions are studies conducted by Onwezen and colleagues that identified a positive relationship between positive emotions and pro-environmental behavior [21, 22]. In samples of Dutch respondents, the authors found that anticipated pride mediated the effects of normative attitudes concerning environmentally friendly behavior on pro-environmental behavioral intentions, such as intending to buy environmentally friendly products [21].

Other recent work suggests a potentially stronger effect of positive compared to negative anticipated emotions on pro-social behavior more generally. In a study on adolescent’s anticipated pride or guilt in situations of moral or immoral actions, Krettenauer and Johnston [25] found an asymmetry between moral emotion expectancies for actions versus inactions: positive emotion expectancies were stronger when anticipating prosocial actions as compared to resisting antisocial impulse, while negative emotion expectancies were stronger when anticipating antisocial behavior as compared to the failure to act pro-socially. Thus, in the context of motivating prosocial behavior, positive emotion expectancies from action (i.e. pride) may be more effective compared to negative emotion expectancies from inaction (i.e. guilt). This finding was confirmed across cultures [26]. Finally, in a recent experience sampling study on daily experiences of transitory moral emotions and pro-environmental behavior among college students, Bissing-Olson, Fielding and Iyer [20] found that feelings of pride, but not guilt, led to subsequent self-reported pro-environmental behaviors. However, this relationship was found only among participants who perceived positive pro-environmental descriptive norms among their peers. Although the authors did not experimentally test the role of anticipated emotions and are thus unable to make claims of causality, these results point to the potentially more powerful role that anticipated pride could play in motivating green (environmentally friendly) behavior compared to anticipated guilt. A logical next step would be to experimentally test and compare the roles of anticipated pride versus guilt on pro-environmental motivation.

### Present research

Taken together, previous findings suggest that in the environmental domain—a domain that is generally perceived as being impersonal and distant [27] yet also emotionally and morally engaging [23, 15]—anticipated emotions may be powerful leverage points for promoting pro-environmental motivation. Moreover, the small number of studies that have investigated the role of anticipated guilt and pride (or negative and positive emotions more generally) indicate that in this domain, positive anticipated emotions may exert a stronger “pull” than the “push” provided by anticipating negative emotions. One important shortcoming of extant research in this domain, however, is that past studies of anticipated emotions and pro-environmental behavior (that we have been able to identify) are correlational in design and treat emotions as factors which affect behavior indirectly rather than as direct causal drivers of pro-environmental motivation and behavioral intent. This is problematic for applying these findings in practice, as correlational results may not directly translate into effective intervention strategies. Indeed, there may be a difference in efficacy between an intervention that explicitly aims to make certain emotions salient versus one that indirectly causes certain emotional responses.

Further, although some past work demonstrates a positive relationship between anticipated guilt and pro-environmental behavior, it seems plausible that heavy-handed interventions that explicitly target negative feelings will result in reactance and subsequent decreases in desired behavior [28]. Indeed, there is a growing realization that messaging that targets negative emotions, if expressed too drastically, may lead to anger and reactance and actually discourage desired behavioral outcomes [17–19]. Targeting negative emotions may also lead to a single action bias [29, 27], whereas positive emotions are more likely to produce a virtuous cycle of pro-social behavior [30].

The objective of the present study then, is twofold: first, to assess whether making positive and negative anticipated emotions salient just prior to decision making can influence pro-environmental decision making; and second, to determine whether targeting positive anticipated emotions (i.e., pride) is relatively more effective than targeting negative anticipated emotions (i.e., guilt). As discussed above, past work in this domain has only examined the mediating effects of anticipated emotions on pro-environmental motivation or the influence of currently experienced emotions on subsequent behavior, rather than experimentally manipulating the salience of anticipated emotions prior to decision making. The present research thus provides a critical missing piece to our understanding of the role anticipated emotions play for pro-environmental decision making.

In order to pursue these core research objectives, we examine the causal influence of anticipated pride versus guilt on pro-environmental decision making and behavioral intentions using an experimental design. This allows us to clarify the relative impact of anticipating pride from an environmentally friendly (green) choice versus guilt from a non-environmentally friendly (brown) choice on environmental decisions. Specifically, we induced participants to reflect upon the future feelings of pride or guilt they might feel as a result of making or not making particular pro-environmental choices, prior to providing opportunities for them to engage in a range of environmental decisions. Given the past findings reviewed above, we hypothesized that pro-environmental choices will be greater among individuals induced to anticipate feelings of pride relative to those induced to anticipate feelings of guilt. Testing this hypothesis is of crucial interest to our primary research question, namely, how the emphasis of anticipated pride versus guilt differentially impacts environmental decision making.

### Pilot

A pilot study tested whether making an environmental choice would lead to discrete emotional responses. Results from the pilot study confirmed our expectations that feelings of guilt accompany or follow from making a non-environmentally friendly choice, while the feeling of pride accompanies or follows from a pro-environmental choice. These relationships provide the basis for studying anticipated pride and guilt in their influence on pro-environmental decision making. The pilot also allowed for the development of sensitive measures of environmental decision making used in the subsequent experiment. A diverse sample of 545 U.S. participants were randomly presented with three out of ten possible environmental choice scenarios, each of which asked them to make a binary decision between a ‘green’ (pro-environmental) option and a ‘brown’ (non-environmentally friendly) option. Results revealed a positive correlational relationship between the experienced level of pride and the likelihood of choosing the green option, such that higher levels of experienced pride were associated with a higher likelihood of choosing green. Conversely, we found a negative relationship between the experienced level of guilt and the likelihood of choosing the green option, controlling for a range of demographic variables as well as environmental attitude. A full description of the pilot methods and results are included in the supplementary materials. Building on these pilot results, the experimental study examined the causal effects of anticipated pride versus guilt on pro-environmental decision making and behavioral intentions by making anticipated emotions (i.e. pride and guilt) salient just prior to making a series of environmental decisions.

## Method

A diverse sample of 1,050 U.S participants was recruited through Amazon’s Mechanical Turk to participate in an online study. An estimate of target sample size was determined using results from similar studies run in our laboratory using MTurk workers to provide initial estimates of effect sizes for studies of this type, which suggested a small to medium effect size was likely [31, 32]. To ensure data quality, incomplete responses as well as participants having taken longer than three standard deviations above the mean or shorter than 1/3^rd^ of the median experimental completion time (which was positively skewed) were removed. Final sample size for analysis purposes was 987 participants. This data exclusion and cleaning approach did not change the overall pattern of results, which remained the same when all participants were included. The study was reviewed and approved by Columbia University’s Institutional Review Board. All participants gave informed consent to participate in the study.

Participants were asked to reflect upon either (1) the future pride they might feel as a result of taking a particular pro-environmental action, or (2) the future guilt they might feel as a result of not taking a particular pro-environmental action, prior to making a series of environmental decisions. Participants were randomly exposed to one of three induction methods outlined below, which existed in two versions (pride or guilt), and were thus assigned to one of 6 treatment groups in a 2 (guilt vs. pride) x 3 (induction type) design. The three different induction methods were chosen to ensure that observed effects did not merely depend on one specific type of anticipated emotion induction, but generalized across a broad range of induction methodologies from prior literature.

The *one sentence reminder (OS)* was the briefest and most direct of all inductions used. It consisted of a single sentence, modeled after Connolly, Reb and Kausel [33], which was displayed on the top of the screen while participants completed the survey questions: “*As you make your decisions*, *keep in mind that you might feel proud [guilty] about your decisions and the alternatives you have picked*.” Participants exposed to the *affective forecasting (AF)* induction were asked to consider feelings of anticipated pride or guilt associated with environmental choices. Specifically, participants were presented with five green versus brown choice scenarios, similar to the ones used in the pilot study. To induce participants to consider anticipated pride or guilt, they were asked to read each scenario and to imagine how proud [guilty] they would feel in the future if they selected (hypothetically) the green [brown] option. Finally, participants exposed to the *writing prompt (WP)* induction were asked to write a brief essay in which they reflected upon a real upcoming decision in which their future choice would make them feel proud or guilty. To help participants think of a future decision to reflect upon, several examples were provided, such as deciding [*not*] to donate blood or deciding [*not*] to help a friend move. The online supplementary materials provide a complete description of all three induction methods. We also included a control group to provide baseline data on the behavioral measures in this population. Participants in this condition were only exposed to the outcome measures, as described below.

As a manipulation check to confirm that the anticipated emotion inductions increased levels of anticipated pride or guilt, at the end of the experiment, participants were asked to report the strength of the anticipated pride or guilt they considered while making environmental decisions. Specifically, participants were asked to give ratings for anticipated pride, guilt, and a range of other emotions, which served as fillers to distract from the two anticipated emotions of interest. Note that the manipulation check was designed to test the effectiveness of our experimental inductions, and was not intended to be used for mechanistic analysis or to compare the relative strength of the anticipated emotion inductions. We did not expect anticipated pride and guilt levels to be reported at similar levels across experimental conditions, as the motivation to report positive versus negative emotions may differ. Participants may exhibit a tendency to under-report feelings of guilt in order to avoid self-discrepancies [34] and over-report feelings of pride to maintain a positive self-image and self-integrity [35–37]. Rather, we expected that anticipated pride would be higher in the pride conditions than in the guilt conditions, and that anticipated guilt would be higher in the guilt conditions than in the pride conditions.

After being exposed to one of the anticipated pride or guilt inductions, all participants were asked to make five environmental decisions. First, participants were exposed to a hypothetical choice scenario involving a social trade-off. Participants were asked to choose between a ‘green’ (pro-environmental) option and a ‘brown’ (non-environmentally friendly) option. The green option was a sofa made out of sustainable and environmentally friendly bamboo fabric, which was only available in somewhat outdated styles. The brown option was a sofa produced using non-sustainable and non-environmentally friendly bleaches, chemicals and synthetic fabrics but coming in more modern styles. Pre-testing of the choice scenario in the pilot study had revealed a roughly 50/50 split between green and brown choices ensuring that there would not be floor or ceiling effects. Participants were next presented with a hypothetical ‘opt-in’ scenario [38] in which they could choose as few or as many of 14 green amenities for their apartment, such as an energy-star rated fridge. Each amenity added $3 extra per month to their rent. A higher number of chosen environmental amenities indicated increased pro-environmental engagement. Participants then responded to two measures assessing their pro-environmental behavioral intentions. The first measure assessed future consumer choice behavior and consisted of one question item, which asked participants about their likelihood of “buying a green product in the next month” (1 = *not at all likely*, 7 = *extremely likely*). The second measure used the average score of six items that asked participants how often they intended to perform a series of sustainable actions over the next month, including “unplug appliances and chargers at night” [31]. In addition to measuring behavioral intentions, we included a consequential measure of actual behavior: people’s willingness to invest in environmental sustainability [31]. We gave participants the option of donating part of a potential $10 bonus, as determined by lottery, to make a real financial donation to a nonprofit environmental advocacy organization, *Trees for the Future*. Participants typed in the amount they would donate, from $0 to $10. One participant was randomly selected to receive the bonus, and the indicated donation amount was given to the organization. The online supplementary materials provide a complete description of all outcome measures.

## Results

We used a 2 (anticipated emotion: pride vs. guilt) x 3 (induction type: OS vs. AF vs. WP) between-participants factorial design, with separate models (ANOVAs, using a type III partition for the sums of squares) for each of the continuous dependent variables (opt-in, behavioral intentions, donation) and logistic regression for the categorical dependent variable (social choice scenario). To test that effects did not differ across induction methods, the models assessed the effects of the three induction methods, as well as differences in the effect of anticipated pride versus guilt, as well as potential interactions between induction method and the two types of anticipated emotions. As expected, analyses did not reveal a consistent main effect of induction method nor consistent interactions between emotional treatment and induction method, suggesting that observed effects should not depend on one specific induction method. A full report of these statistical analyses is described in the supplementary materials. Results are collapsed across the three induction methods.

Predicted patterns emerged for self-reported ratings of anticipated pride and guilt among participants who were exposed to the pride or guilt inductions, as revealed in the manipulation check. As predicted, participants exposed to an anticipated pride induction reported higher ratings of anticipated pride considered during the decision making process compared with participants who anticipated feeling guilt (anticipated pride rating, pride induction group, *M* = 4.13, *SD* = 1.9; anticipated pride rating, guilt induction group, *M* = 3.59, *SD* = 2.0, *t*(843.77) = -3.99, *p* < .001; Cohen’s *d* = .27). Relatedly, those exposed to an anticipated guilt induction reported higher ratings of anticipated guilt compared with those exposed to an anticipated pride induction (anticipated guilt rating, guilt induction group, *M* = 2.51, *SD* = 1.57; anticipated guilt rating, pride induction group, *M* = 2.13, *SD* = 1.47, *t*(843.29) = 3.68, *p* < .001; Cohen’s *d* = .25).

As an additional manipulation check for the writing prompt inductions, a content analysis of participants’ pride and guilt essays was conducted using the Linguistic Inquiry and Word Count software [39]. As expected, results from the content analysis showed that participants in the anticipated pride condition used significantly more positive affect words compared to participants in the anticipated guilt condition, who used more negative affect words (percent positive affect words in anticipated pride essay, *M* = 5.88, *SD* = 2.64; percent positive affect words in anticipated guilt essay, *M* = 2.54, *SD* = 1.92; *t*(237.73) = 11.95, *p* < .001; Cohen’s *d* = 1.46; percent negative affect words in anticipated guilt essay, *M* = 3.69, *SD* = 2.37; percent negative affect words in anticipated pride essay, *M* = 0.69, *SD* = 1.11; *t*(206.71) = -13.64, *p* < .001; Cohen’s *d* = 1.6).

**Fig 1:**
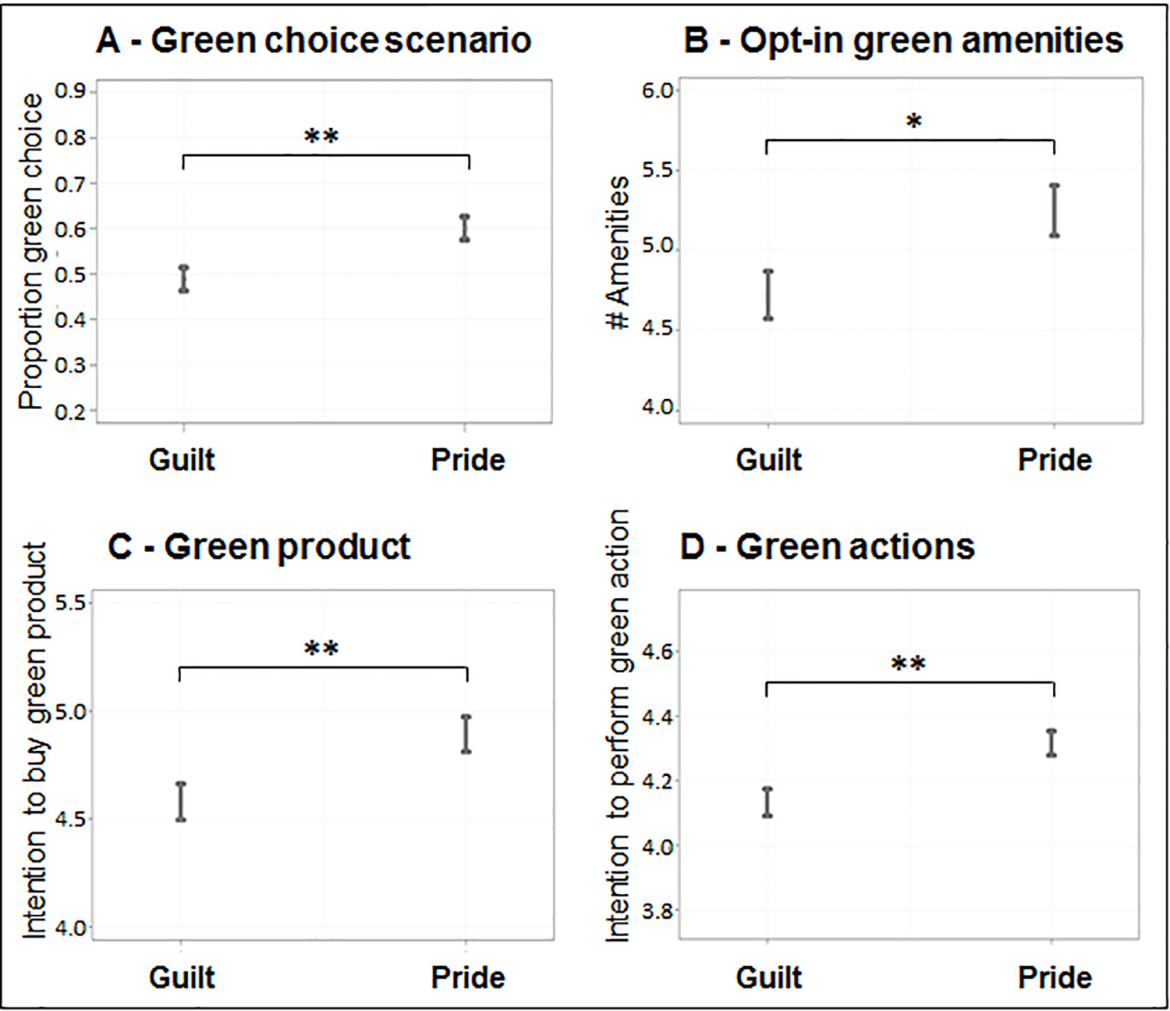
Influence of pride and guilt inductions on pro-environmental behavior per outcome measure. Fig 1 depicts four out of all five outcome measures that show a significant difference in pro-environmental behavior between those induced with pride and those induced with guilt. (A): Analysis of Deviance (Type III partition of the sums of squares); (B)-(D): Analysis of variance (Type III partition of the sums of squares). Y-axes: (A) = proportion of green choice; (B) = number of amenities chosen, range 0 to 14; (C) = intention to buy green product over next month, range 1 (not at all likely) to 7 (extremely likely); (D) = how often participant intends to perform a series of pro-environmental actions, range 1 (never) to 6 (all the time); (A): error bars denote binomial approximation of the standard error; (B)-(D): error bars denote standard error; Donation (not depicted): *F* = .23, *p* = .632.

Results revealed a main effect of anticipated emotion (pride vs. guilt) across four of the five measures of environmental decision making. Fig 1 depicts the results of the aggregated anticipated pride induced groups and aggregated anticipated guilt induced groups, respectively. Overall, participants who were exposed to an anticipated pride induction consistently reported higher pro-environmental intentions compared to those exposed to an anticipated guilt induction. Compared to participants who anticipated guilt, those exposed to the anticipated pride induction were more likely to choose the green option in the choice scenario, *β* = -0.23, *z*(844) = -3.28, *p* = .001; LR *X*^2^ (1) = 10.83, *p* < .001; select more green amenities for their apartments, *F* (1, 845) = 5.99, *p* = .015; were more likely to intend to buy a green product over the next month, *F*(1,844) = 7.17, *p* = .008); and intend to perform a range of green actions, as compared to the participants induced to anticipate guilt, *F*(1,845) = 10.58, *p* = .001. Donation behavior did not reveal a similar pattern: Participants induced to anticipate pride did not donate significantly more than participants induced to anticipate guilt, *F*(1,844) = 0.23, *p* = .632.

**Fig 2:**
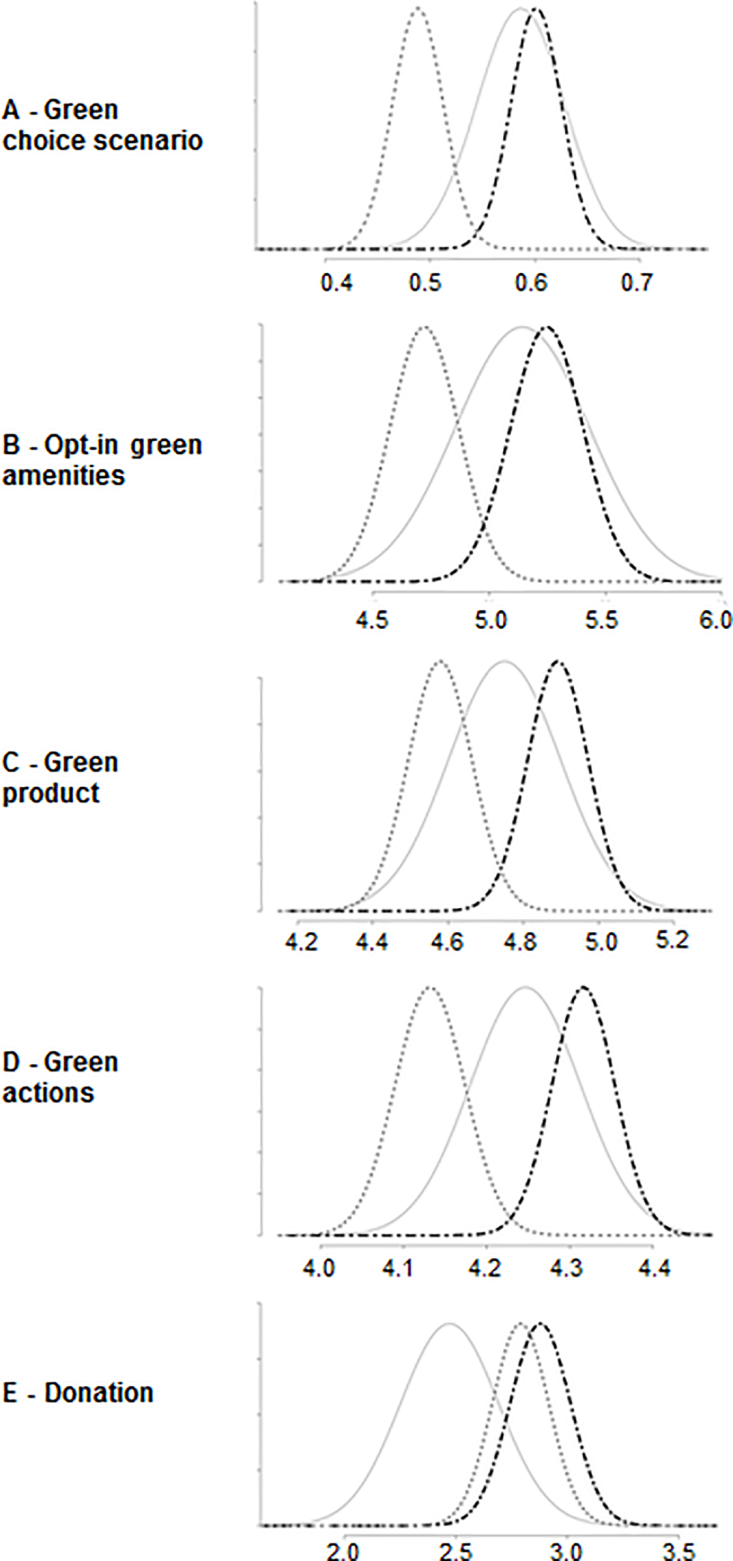
Density plots of anticipated pride, guilt, as well as control group distributions. Y-axis: probability density; X-axis: (A) = proportion of green choice; (B) = number of amenities chosen, range 0 to 14; (C) = intention to buy green product over next month, range 1 (not at all likely) to 7 (extremely likely); (D) = how often participant intends to perform a series of pro-environmental actions, range 1 (never) to 6 (all the time); (E) = donation amount, range $0 to $10; legend: solid grey line = control group distribution, dotted dark grey line = guilt induction group distribution, black doted-dashed line = pride induction group distribution.

Analysis of the control group revealed an interesting pattern. Across all four dependent measures of pro-environmental behavioral intention, descriptive results for the control group fall between those of the anticipated pride and guilt groups, with behavioral intentions for the anticipated pride group being higher than the control group, and behavioral intentions for the anticipated guilt group being lower than the control group. For the donation dependent measure, both anticipated pride and guilt groups lie above the control group, with the anticipated pride group being higher than the anticipated guilt group. Fig 2 depicts the distributions for anticipated pride, anticipated guilt, as well as control groups.

These observed descriptive trends did not reach statistical significance. There was no statistically significant difference between the proportion of green choices in the choice scenario between the pride group (proportion green choices = .6) and control group (proportion green choices = .59; *X*^2^ (1) = .05, *p* = .827) or the guilt group (proportion green choices = .49) and the control group (*X*^2^ (1) = 3.63, *p* = .057). Likewise, there was no statistically significant difference between the number of green amenities chosen between the pride group (*M* = 5.25, *SD* = 3.18) and the control group (*M* = 5.14, *SD* = 3.41; *t*(226.24) = -.32, *p* = .749) or the guilt group (*M* = 4.72, *SD* = 3.07) and the control group (*t*(217.04) = 1.3, *p* = .194). Intentions to buy a green product over the next month did not significantly differ between the pride group (*M* = 4.89, *SD* = 1.64) and the control group (*M* = 4.75, *SD* = 1.76; *t*(225.3) = -.84, *p* = .403) or the guilt group (*M* = 4.58, *SD* = 1.75) and the control group (*t*(234.99) = 1.0, *p* = .32); neither did intentions to perform a range of green actions (pride group, *M* = 4.32, *SD* = .77, control group, *M* = 4.25, *SD* = .79; *t*(235.18) = -.91, *p* = .363); guilt group, *M* = 4.13, *SD* = .87; *t*(259.33) = 1.46, *p* = .146). Finally, donations did not significantly differ between the pride group (*M* = 2.88, *SD* = 2.76) and the control group (*M* = 2.47, *SD* = 2.6; *t*(252.32) = -1.58, *p* = .1 16), nor between the guilt group (*M* = 2.79, *SD* = 2.62) and the control group (*t*(237.89) = -1.26, *p* = .21).

## Discussion

The present work deepens and expands existing knowledge regarding the relationship between anticipated emotions and pro-environmental decision making. We find evidence that there are distinct differences in the effect on pro-environmental behavioral intentions when inducing people to anticipate the pride they would feel related to pro-environmental action compared to the guilt they would feel from inaction. Notably, inducing people to anticipate feelings of pride for positive future actions appears to have a more powerful effect on pro-environmental motivation compared to prompting feelings of guilt for inactions. This core finding not only contributes to the literature by disentangling the divergent effects of anticipated pride and guilt on pro-environmental decision making and motivation, but it also challenges current pro-environmental messaging strategies, which favor inducing negative emotions such as guilt to promote mitigation behaviors. Guilt-arousing communications have received considerable attention in extant research, particularly in the context of green marketing communications, [40–43]. Guilt appeals are commonly used by social marketers as a persuasion tactic [44, 40, 45, 46, 7, 47]. In fact, a content analysis of emotional appeals used for persuasion revealed that guilt appeals are used at a level comparable to that of other communication strategies (such a humor or sexual) and are most often employed by charities or health-related products [48].

Our results suggest that guilt arousing communications may be less effective in the domain of environmental conservation compared to pride arousing communications, at least in some contexts. How do we explain the relative advantage of anticipating pride over guilt that we observed in this study? One obvious explanation comes from the robust literature on reactance [28], which suggests that people respond poorly when told to feel badly about themselves for some perceived moral failing [49]. Although the anticipation of guilt, when it happens, is indeed likely to result in positive behavior [14, 50], it seems likely that many efforts to induce such feelings fall flat or perhaps even boomerang, resulting in a lack of behavior change or possibly even retaliatory behavior. In line with this account, recent work has shown that negative affect-based messaging can lead to a decrease in pro-environmental spillover behaviors, i.e. a decreased likelihood of additional pro-environmental behaviors beyond the targeted ones [51]. Taeuber, Van Zomeren and Kutlaca [52] review evidence that negative moral framing of persuasive messages, such as guilt frames, may constitute a threat to a person’s self-image and morality, and may hence lead to defensive reactions instead of the intended positive behavioral outcomes. In contrast, individuals are likely much less motivated to react poorly to inducements to feel proud about themselves, and thus the anticipation of such positive emotions is more likely to actually take root (and in turn motivate positive behavior).

A related body of research has explored the moderating conditions under which reactance may occur in the field of guilt appeals, which suggest that our results could be driven by the situational context of the decision. For example, Bessarabova and colleagues [53] suggest that the effect of guilt on reactance is mediated through the explicit awareness that messages use guilt to induce persuasion, which makes people infer an overt persuasive attempt regardless of the source’s intention. It is possible that our explicit anticipated guilt inductions contributed to feelings of reactance, but that more subtle anticipated guilt primes would more effectively promote pro-environmental action. Other work indicates that advantageous effects of guilt appeals are found when promoting a highly proximal issue to people with low levels of environmental consciousness [54] or when including the presence of reparation suggestions [55]; contextual features that were not present in our own inductions. Further studies explicitly testing the process of guilt-induced reactance will be useful in illuminating the underlying conditions under which an anticipated guilt induction triggers reactance. Although our evidence is indirect with respect to the moderators at work, our results confirm our hypothesis that inducing anticipating feeling proud of one’s future mitigation efforts has a more positive effect on pro-environmental motivation relative to inducing anticipating feeling guilty for inaction.

In line with the presented literature, results comparing emotional induction effects to baseline behavior point towards a potentially reaction arousing effect of guilt appeals. While pride may increase target behavior above baseline behavior, guilt may dampen behavior below baseline levels. Although these results did not reach statistical significance, these trends are in line with the notion that guilt appeals may lead to reactance and subsequent decreases in desired behavior. Our study was designed and appropriately powered to detect differences between anticipated pride and guilt conditions as this comparison constitutes the main focus of this research. We included the control group to better understand how the anticipated pride and guilt groups relate to a baseline. Further research focusing on the baseline comparison is needed to investigate the observed relationships in more detail. Our current results suggest that pro-environmental messaging and campaigns that do utilize an emotional appeal should probably aim to induce anticipation of positive emotions rather than anticipation of negative emotions.

Our research also highlights a critical yet often unrecognized (within this domain, at least) distinction between treating emotional experiences as *antecedents* to behavior versus as *consequences* of action. Because extant literature in this domain has treated emotions nearly exclusively as mediating and moderating factors, investigating indirect effects on behavior rather than as causal antecedents to behavior, past work has not been able to identify the divergent effects of anticipating pride versus guilt on pro-environmental motivation. While people may experience guilt in response to making a non-environmentally friendly decision, as we show in our pilot study and while in theory these emotional experiences could be leveraged to promote sustainable behavior through the motivation to avoid experiencing guilt, it seems that trying to induce such considerations leads to lower levels of pro-environmental motivation compared to inducing pride considerations. Moreover, and perhaps even more damagingly in the long run, an incomplete understanding of how positive and negative emotions differentially motivate behavior has, it seems, led many advocates to the belief that negative emotions such as guilt are the more powerful levers for pro-environmental behavior change. Our results provide indications that this may not be the case and point to the urgency of investigating the effects of the use of anticipated emotions in environmental messaging further to avoid potential negative rebound effects. Further research should also test whether such interventions produce sustainable changes in actual behaviors over time. Indeed, changes in attitudes and self-reported behavior may not necessarily correspond to behavior change, depending on how closely the attitude in question corresponds to a target behavior [56]. In future work, these issues could be more thoroughly explored through the use of additional methodological frameworks, including in-person studies where real environmental actions, such as recycling behavior, can be observed.

## Conclusion

The question of what shapes pro-environmental behavior is complex, and strategies are needed to engender more intrinsic motivations through the use of experiential and psychological factors, including emotional response [57]. Our work presents novel findings on the differential role of two specific anticipated emotions in shaping pro-environmental decision making and motivation, revealing important practical implications and opening up avenues for future research on effective intervention design. Our results contribute to efforts to clarify and disentangle the differential roles anticipated pride versus guilt play in shaping pro-environmental decision making. Findings may translate to other domains of pro-social behavior of public and applied interest as well, such as health behaviors or providing humanitarian aid. Our results point to a more beneficial role of anticipated pride compared to guilt in shaping pro-environmental motivation and furthermore highlight the need for careful assessment of communication and messaging strategies that employ emotional appeals, as effects may vary substantially depending on the emotion targeted. Policy makers, advocacy organizations and others would benefit from a more nuanced understanding of the impact of induced anticipated emotions on pro-environmental decision making and motivation, to leverage positive effects and avoid potential negative ones. Our results provide a first step towards this worthy end.

## Supporting information

S1 File Additional description of results, demographic characteristics of the study sample, induction methods, and all outcome measures of the experimental study. (PDF)

S2 File Additional description of methods, results, demographic characteristics of the study sample, and list of choice scenarios of the pilot study. (PDF)
