# Supplementary material for: The influence of anticipated pride and guilt on pro-environmental decision making
Source: PLoS One. 2017 Nov 30;12(11):e0188781. doi: 10.1371/journal.pone.0188781 (PMC5708744; doi:10.1371/journal.pone.0188781)
Supplement: S1 File — (PDF) [file pone.0188781.s001.pdf]

Supplementary Materials:

The influence of anticipated pride and guilt on pro-environmental decision making

PLOS ONE

Claudia R. Schneider*, Lisa Zaval, Elke U. Weber & Ezra M. Markowitz

*Corresponding author affiliations:

Department of Psychology, Columbia University, New York, NY, USA.

Center for Research on Environmental Decisions, Columbia University, New York, NY, USA.

Center for Decision Sciences, Columbia University, New York, NY, USA.

Corresponding author email: crschneider@psych.columbia.edu

S1 File. Additional description of results, demographic characteristics of the study sample, induction methods, and all outcome measures of the experimental study.

Content

S1. Experimental study
- S1.a. Results
  - S1.a.i Statistical analysis anticipated emotion x induction type
- S1.b Tables
  - S1.b.i Demographic characteristics of the study sample
- S1.c Measures
  - S1.c.i Description of induction methods
  - S1.c.ii Description of all outcome measures

S1.a. Experimental study Results

S1.a.i. Statistical analysis anticipated emotion x induction type

As described in the manuscript, we used a 2 (anticipated emotion: pride vs. guilt) x 3 (induction type: OS vs. AF vs. WP) between-participants factorial design, with separate models (ANOVAs, using a type III partition for the sums of squares) for each of the continuous dependent variables (opt-in, behavioral intentions, donation) and logistic regression for the categorical dependent variable (social choice scenario). To test that effects did not differ across induction methods, the models assessed the effects of the three induction methods, as well as differences in the effect of anticipated pride versus guilt, as well as potential interactions between induction method and the two types of anticipated emotions. As expected, analyses did not reveal a consistent main effect of induction method nor consistent interactions between emotional treatment and induction method, suggesting that observed effects should not depend on one specific induction method. Please refer to the ANOVA tables below for detailed statistical results.

A – Green choice scenario:

	df	LR X2	p
induction type	2	4.64	.1
emotion	1	11.05	< .01
induction type * emotion	2	.17	.92

B – Opt-in green amenities:

	df	SS	F	p
induction type	2	35.1	1.81	.16
emotion	1	55	5.68	.02
induction type * emotion	2	71.8	3.7	.03

C – Green product:

	df	SS	F	p
induction type	2	2.5	.43	.65
emotion	1	20.5	7.09	< .01
induction type * emotion	2	.4	.07	.94

D – Green actions:

	df	SS	F	p
induction type	2	6	4.43	.01
emotion	1	7.2	10.75	< .01
induction type * emotion	2	1.1	.81	.44

E – Donation:

	df	SS	F	p
induction type	2	58	4.03	.02
emotion	1	2.1	.29	.59
induction type * emotion	2	13.8	.96	.38

S1.b. Experimental study supplementary Tables

Table S1.b.i. Demographic characteristics of the study sample

Variable	**Experimental study** (N = 987)
Gender, %	
Females	46.81
Males	52.68
Age, *M* (*SD*)	34.82 (11.1)
Education, %	43.06
Income, %	40.02
Race/ethnicity, %	
White	73.45
Polit. Affiliation, %	
Democrat	42.96
Republican	14.39
Independent/Other	42.55

Note: Due to some participants choosing not to answer, the gender, race/ethnicity, political affiliation columns do not total to 100.

*Educational Attainment = *at least some college.*

*Household income in 2013 (Pilot study) / 2014 (Experimental study) = *$50,000 or over*

All participants had a 97% or higher approval rating according to the screening procedures of Amazon’s Mechanical Turk.

S1.c. Experimental study Measures

S1.c.i. Description of induction methods

One sentence reminder (OS):

Pride:
As you make your decisions, keep in mind that you might feel proud about your decisions and the alternatives you have picked
Guilt:
As you make your decisions, keep in mind that you might feel guilty about your decisions and the alternatives you have picked.

Affective forecasting (AF):

Scenario 1:
Imagine that you are the head of a small start-up company that is deciding whether or not to enact environmentally friendly practices. These include providing recycling bins throughout the office and introducing a reusable mug policy in the staff kitchen area. Recycling reduces waste that has to be burned and conserves resources. Reusable mugs avoid the use of Styrofoam cups which are bad for the environment since they never decompose. However, if you choose to enact green practices you have to stay 10min longer at the end of the day each day to ensure that the dishwasher is loaded and started and you have to get to work 15min earlier every day to make sure that the recycling bins are set up properly and to unload the dishwasher. If you stick with the regular trash bins and the Styrofoam cups in the staff kitchen, you would not have to spend the extra time.

Pride:
Imagine you choose to enact green practices by providing recycling bins and introducing a reusable mug policy. How proud would you feel after having made this decision?

9-point scale: not at all proud - extremely proud

Guilt:
Imagine you choose to stick with using regular trash bins and Styrofoam cups. How guilty would you feel after having made this decision?

9-point scale: not at all guilty - extremely guilty

Scenario 2:
Imagine that you are buying a new car. You could buy an environmentally friendly, highly fuel-efficient car (such as a hybrid vehicle), which is much better for the environment, and reduces local air pollution by reducing vehicle emissions. However this type of vehicle is also significantly more costly. Or you could purchase a cheaper but less fuel efficient vehicle, freeing up that money to be put to other "better" uses right away.

Pride:
Imagine you choose the hybrid vehicle. How proud would you feel after having made this decision?

9-point scale: not at all proud - extremely proud

Guilt:
Imagine you choose the cheaper, less fuel-efficient vehicle. How guilty would you feel after having made this decision?

9-point scale: not at all guilty - extremely guilty

Scenario 3-5:
For the next set of questions, we want you to consider that you are shopping for three products: (a) a dishwasher, (b) a household cleaner, and (c) a backpack.

Scenario 3:
Imagine that you are out shopping for a dishwasher, and you are choosing between two kinds. Below are brief descriptions of the two products that you are choosing between. Please read them carefully.

PRODUCT A

Sub-Zero ED40 Elite Dishwasher ($1,100)

- Comes in choice of stainless steel or white exterior with black chrome trim
- Features a revolutionary heated drying system that eliminates water spots
- Has powerful water sprays but produces no sound

PRODUCT B

Sub-Zero Eco-Friendly Dishwasher ($1,100)

- Has a standard 40-minute running cycle
- Uses a recirculating water system to save water
- Is made with recycled components

Pride:
Now imagine that you choose to select the Sub-Zero Eco-Friendly Dishwasher. Imagine how proud you would feel having made this decision. On the scale below, indicate the level of pride you predict you would feel.

9-point scale: not at all proud - extremely proud

Guilt:
Now imagine that you choose to select the Sub-Zero ED40 Elite Dishwasher. Imagine how guilty you would feel having made this decision. On the scale below, indicate the level of guilt you predict you would feel.

9-point scale: not at all guilty - extremely guilty

Scenario 4:
Imagine that you are out shopping for a household cleaner, and you are choosing between two types. Below are brief descriptions about the two cleaners that you are choosing between. Please

read them carefully.

PRODUCT A	PRODUCT B
**Lysol Industrial Strength Household Cleaner ($7)**	**Lysol Natural Household Cleaner ($7)**
• Awarded most effective cleaner on the market award<br>• Chemically engineered to cut through the toughest grease, rust and mold<br>• Kills 99.9% of germs on contract	• Made from biodegradable nontoxic materials<br>• Contains no acids, dyes, or harsh chemicals<br>• Not tested on animals

Pride:
Now imagine that you choose to select the Lysol Natural Household Cleaner. Imagine how proud you would feel having made this decision. On the scale below, please indicate the level of pride you predict you would feel.

9-point scale: not at all proud - extremely proud

Guilt:
Now imagine that you choose to select the Lysol Industrial Strength Household Cleaner. Imagine how guilty you would feel having made this decision. On the scale below, please indicate the level of guilt you predict you would feel.

9-point scale: not at all guilty - extremely guilty

Scenario 5:
Imagine that you are out shopping for a backpack, and you are choosing between two kinds. Below are brief descriptions of the two backpacks that you are choosing between. Please read them carefully.

PRODUCT A	PRODUCT B
**North Face Ultra-Strength backpack ($64)**	**North Face Eco-Life backpack ($64)**
• Contains eight different storage compartments for maximum versatility<br>• Stylish design crafted with water-resistant coating<br>• Solid construction lasts twice as long as the next leading brand on the market	• Made from 100% organic and recycled fibers<br>• Utilitarian design minimizes waste in the construction process<br>• Comes with instructions on how to recycle the backpack when you are done with it

Pride:
Now imagine that you choose to select the North Face Eco-Life backpack. Imagine how proud you would feel having made this decision. On the scale below, please indicate the level of pride you predict you would feel.

9-point scale: not at all proud - extremely proud

Guilt:
Now imagine that you choose to select the North Face Ultra-Strength backpack. Imagine how guilty you would feel having made this decision. On the scale below, please indicate the level of guilt you predict you would feel.

9-point scale: not at all guilty - extremely guilty

Writing task (WT):
Pride:
How proud would you feel?
For this writing task, we would like you to think about a decision in the future where your choice could make you feel proud. This real decision should occur in the next few months. Examples of decisions that might make you feel proud include deciding to donate blood, deciding to volunteer in a soup kitchen, or deciding to do someone a big favor like helping a friend move. Please think carefully about this. Then, in the space below, please write a brief essay describing the pride you would feel for having made a certain decision. Please tell us about the decision you have in mind and how proud you might feel about the decision once you have made it. Why do you think you would feel proud? This essay should take you approximately 5 minutes to complete.
Please write your response in the space provided below:

Guilt:
How guilty would you feel?
For this writing task, we would like you to think about a decision in the future where your choice could make you feel guilty. This real decision should occur in the next few months. Examples of decisions that might make you feel guilty include deciding not to donate blood, deciding not to volunteer in a soup kitchen, or deciding not to do someone a big favor like helping a friend move. Please think carefully about this. Then, in the space below, please write a brief essay describing the guilt you would feel for having made a certain decision. Please tell us about the decision you have in mind and how guilty you might feel about the decision once you have made it. Why do you think you would feel guilty? This essay should take you approximately 5 minutes to complete.
Please write your response in the space provided below:

S1.c.ii. Description of all outcome measures

A – Social choice scenario:
Imagine you need to purchase new furniture for your home. You are torn between a sofa made out of bamboo fabrics and a sofa made with more traditional fabrics. The bamboo sofa is a sustainable material and very environmentally friendly, however it only comes in somewhat outdated styles. The regular sofa is produced using bleaches, chemicals and synthetic fabrics, but comes in many modern styles. All other relevant factors (such as price, comfort and durability) are the same. Which option would you rather choose: The sofa made out of bamboo fabrics (option A) or the sofa made of traditional fabrics (option B)?

❍ Option A
❍ Option B

B – Opt-in green amenities (adapted from Steffel et al., 2015)**:**
Imagine that you are moving into a new apartment complex and have the option of choosing which of 14 environmentally friendly, 'green' amenities that you would like to have installed in your apartment. None of the green amenities are already included in the standard rent, but if you would like any of the green amenities to be installed, the landlord would add a small amount ($3 dollars) to your monthly rent for each amenity that you choose to add. Please check which of the green amenities below you would install in this scenario (you may choose multiple):

❑ Energy-star furnace & air-conditioner
❑ Tankless water-heater
❑ Programmable thermostat
❑ Storm windows and doors
❑ Airflow-adjusting ceiling fans
❑ UV filter film on windows
❑ Energy-efficient dishwasher and refrigerator
❑ Compact Florescent (CFL) light bulbs
❑ Energy-efficient washer & dryer
❑ Dimmer switches for indoor lighting
❑ Low-flow toilets
❑ Solar-powered outdoor lighting
❑ Low-flow faucets & shower heads
❑ Motion sensors for outdoor lighting

C – Intention to buy green product (adapted from Zaval, Markowitz & Weber, 2015)**:**
How likely are you to buy a green (environmentally-friendly) product next month?
7 point scale, not at all likely – extremely likely

D – Intention to perform green actions (α = 0.55; stand. α = 0.57; adapted from Zaval, Markowitz & Weber, 2015)**:**
Please indicate how often you intend to perform the following behaviors over the next month:

	Never	Very infrequently	Once in a while	Sometimes	Often	All the time
Turn off lights whenever leaving a room	❍	❍	❍	❍	❍	❍

Wait until I have a full load before doing my laundry	❍	❍	❍	❍	❍	❍
Recycle used paper at home or work/school	❍	❍	❍	❍	❍	❍
Take showers that are 5 minutes or less	❍	❍	❍	❍	❍	❍
Use public transportation or carpool	❍	❍	❍	❍	❍	❍
Unplug appliances and chargers (e.g., TV, cell phone, computer) at night	❍	❍	❍	❍	❍	❍

E – Donation (adapted from Zaval, Markowitz & Weber, 2015)**:**
As an extra "thank you" for participating in our research today, we will enter you into a lottery to win a $10 bonus. One study participant will be chosen at random to receive this bonus (which will be given to you via MTurk). We also would like to give you an opportunity to donate some or all of the bonus to a charitable organization, if you are the lottery winner. You may split the $10 between yourself and the charity however you want to, using the form on the next page. Any money you allocate to the charity will be directly donated on your behalf by the research team. The charity you may donate to today will be shown on the next page.

The organization you have an opportunity to donate to today is called Trees for the Future, whose motto is "Plant trees. Change Lives." Since 1989, Trees for the Future has helped communities in 19 countries around the world plant millions of trees. Their work has and will continue to improve the well-being of children and families for generations to come, by cleaning the air, reducing risks from landslides and reducing deforestation. If you'd like to learn more about the organization, their website is: http://www.treesforthefuture.org

Please note that the total amount must add up to exactly $10. Remember that you will be paid your MTurk compensation regardless of whether you win the lottery or not.

______ Donate to Trees for the Future

______ Keep for myself
