# Supplementary material for: The influence of anticipated pride and guilt on pro-environmental decision making
Source: PLoS One. 2017 Nov 30;12(11):e0188781. doi: 10.1371/journal.pone.0188781 (PMC5708744; doi:10.1371/journal.pone.0188781)
Supplement: S2 File — (PDF) [file pone.0188781.s002.pdf]

Supplementary Materials:

The influence of anticipated pride and guilt on pro-environmental decision making

PLOS ONE

Claudia R. Schneider*, Lisa Zaval, Elke U. Weber & Ezra M. Markowitz

*Corresponding author affiliations:

Department of Psychology, Columbia University, New York, NY, USA.

Center for Research on Environmental Decisions, Columbia University, New York, NY, USA.

Center for Decision Sciences, Columbia University, New York, NY, USA.

Corresponding author email: crschneider@psych.columbia.edu

S2 File. Additional description of methods, results, demographic characteristics of the study sample, and list of choice scenarios of the pilot study.

Content

S2. Pilot study

S2.a Method
S2.b Results
S2.c Figures and Tables
S2.c.i Graphical display of results
S2.c.ii Regression table with controls
S2.c.iii Demographic characteristics of the study sample
S2.d Measures
S2.d.i List of choice scenarios

S2.a. Pilot study Methods

A diverse sample of 545 U.S. participants, recruited through Amazon's Mechanical Turk, elected to participate in an online survey (see Table S2.c.iii for demographic details). Participants were randomly presented with three out of ten possible environmental choice scenarios, each of which asked them to make a binary decision between a 'green' (pro-environmental) option and a 'brown' (non-environmentally friendly) option. The choice scenarios were presented in writing in the form of brief descriptive paragraphs. The ten scenarios presented trade-offs on a variety of dimensions, including social factors, effort, time, or financial cost (see section S2.d. for a full list of scenario items). For example, the time/environmental trade-off scenario read:

> *Imagine that you need to get your car washed and waxed, and received a free online coupon to do so. You can choose to get the regular wash & wax (option A), or you can choose the eco-friendly wash & wax (option B), which uses less water and uses cleaning products that are organic and do not harm the environment. When you arrive at the car wash, you learn that the regular wash takes 15 minutes, while the eco-friendly wash & wax takes 45 minutes. Which option would you choose: The regular wash (option A) or the eco-friendly wash (option B)?*

After reading the scenario and choosing either the green or brown option, participants were asked to rate how much pride and guilt they felt during the decision making process. Participants were also asked to rate feelings of a range of other emotions (engaged, bored, torn, confident, angry, sad, pleased, regretful, satisfied), which served as fillers to distract from the two emotions of interest. Ratings were made on a 5-point Likert scale anchored from not at all to very strongly.

The relationship between reported pride/guilt ratings and choice outcome type (green vs. brown scenario) was assessed across the 10 choice scenarios. We used a multilevel mixed model approach including random and fixed effects to account for repeated measures within a participant, since each participant completed 3 out of 10 choice scenarios. Reported pride and reported guilt were entered as predictors of choice into two regression models, one using pride and one using guilt. Log odds parameter estimates were converted to probability estimations.

S2.b. Pilot study Results

As expected and consistent with findings by Bissing-Olson, Fielding and Iyer (2016), regression results revealed a positive relationship between the experienced level of pride and the likelihood of choosing the green option, $\beta = 0.66$, Wald's X2 (1) = 109.83, $p < .001$. Higher levels of experienced pride were associated with a higher likelihood of choosing green. Conversely, and as predicted, we find a negative relationship between the experienced level of guilt and the likelihood of choosing the green option, $\beta = -1.23$, Wald's X2 (1) = 109.2, $p < .001$. Figure S2.c.i visualizes these results. Results remained robust after controlling for a range of demographic variables (sex, age, education, political affiliation and income) as well as environmental attitude (see Table S2.c.ii).

S2c. Pilot study Figures and Tables

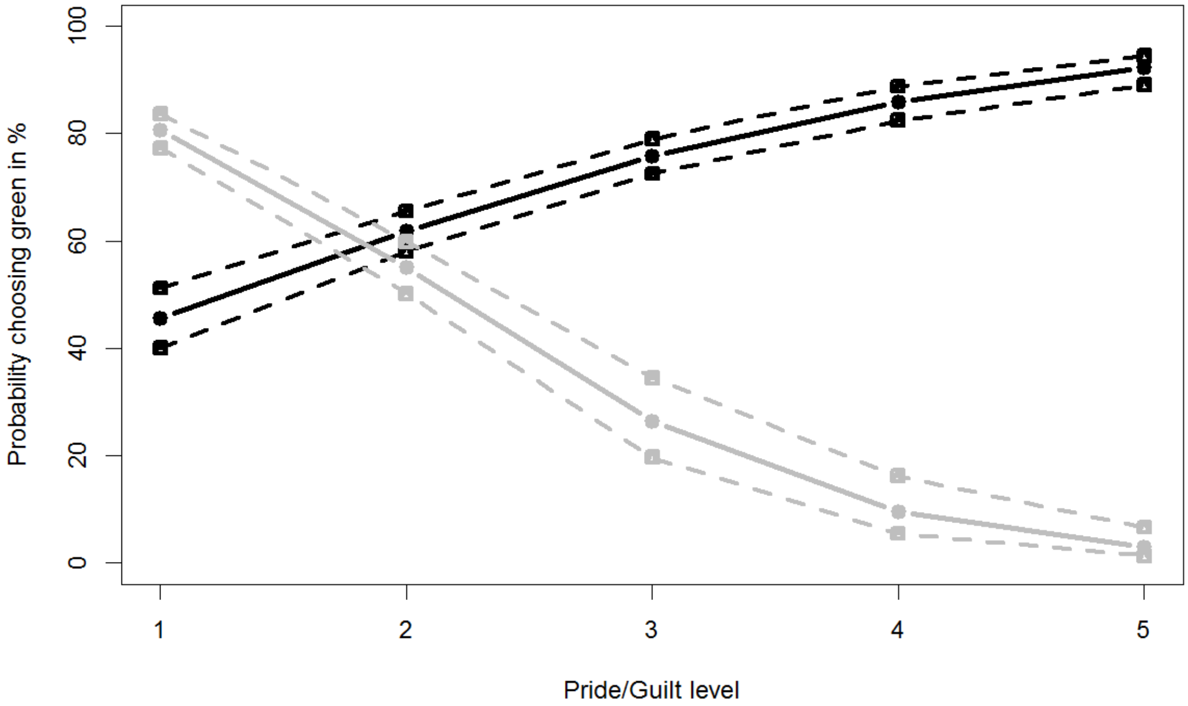

Fig. S2.c.i Relationship between experienced pride[guilt] and green choice; X-axis denotes the 5 levels of the Likert-scale used to indicate pride and guilt levels; black data points = pride, grey data points = guilt; dashed lines denote binomial 95% confidence interval.

Table S2.c.ii: Regression table with controls

All CS		Estimate	Std Error	z value	p-value
1	Pride	0.66	0.06	10.48	< .001 ***
2	Pride	0.55	0.06	9.65	< .001 ***
	Gender	0.26	0.13	2.03	.043 *
	Age	< 0.01	0.01	-0.2	.842
	Education	0.06	0.08	0.78	.437
	Republican (relative to other)	-0.44	0.16	-2.67	.008 **
	Income	-0.14	0.05	-3.20	.001 **
	Care for the environment	0.67	0.08	8.44	< .001 ***
3	Guilt	-1.23	0.12	-10.45	< .001 ***
4	Guilt	-1.10	0.11	-10.10	< .001 ***
	Gender	0.31	0.13	2.30	.022 *
	Age	-0.01	0.01	-1.27	.206
	Education	0.02	0.08	0.22	.823
	Republican (relative to other)	-0.35	0.17	-2.07	.038 *
	Income	-0.10	0.05	-2.25	.024 *
	Care for the environment	0.82	0.08	9.75	< .001 ***

Note: Model 1 + 3 n=545 (1635 observations), Model 2 + 4 n=534 (1602 observations)

Table S2.c.iii. Demographic characteristics of the study sample

Variable	**Pilot study** (N = 545)
Gender, %	
Females	53.03
Males	45.69
Age, *M* (*SD*)	36.83 (11.27)
Education, %	45.14
Income, %	37.43
Race/ethnicity, %	
White	78.35
Polit. Affiliation, %	
Democrat	36.33
Republican	17.25
Independent/Other	46.06

Note: Due to some participants choosing not to answer, the gender, race/ethnicity, political affiliation columns do not total to 100.

*Educational Attainment = *at least some college.*

*Household income in 2013 (Pilot study) / 2014 (Experimental study) = *$50,000 or over*

All participants had a 97% or higher approval rating according to the screening procedures of Amazon’s Mechanical Turk.

S2.d. Pilot study Measures

S2.d.i List of choice scenarios

Scenario 1: Cost1
Imagine that you are the mayor of a major city. Your city council has decided to replace the municipality's fleet of vehicles, which are all old and starting to break down regularly. However, the council is split 50/50 on what type of cars to buy to replace the old ones. Half of the council wants to buy environmentally friendly, highly fuel-efficient cars (such as hybrid vehicles), which are much better for the environment, and reduce local air pollution by reducing vehicle emissions. However they are also significantly more costly. The other half of the council wants to purchase cheaper but less fuel efficient vehicles, freeing up that money to be put to other "better" uses right away, although the specific uses of that money have not yet been determined. At the next council meeting, you will have to cast the deciding vote about which type of car to purchase in replacing the municipality's fleet. Which option will you choose: the hybrid vehicles (Option A) or the regular vehicles (Option B)?

- ❍ Option A
- ❍ Option B

Scenario 2: Cost1Table
Imagine that you are the mayor of a major city. Your city council has decided to replace the municipality's fleet of vehicles, which are all old and starting to break down regularly. However, the council is split 50/50 on what type of cars to buy to replace the old ones. Half of the council wants to buy environmentally friendly, highly fuel-efficient cars (such as hybrid vehicles), which are much better for the environment, and reduce local air pollution by reducing vehicle emissions. However they are also significantly more costly. The other half of the council wants to purchase cheaper but less fuel efficient vehicles, freeing up that money to be put to other "better" uses right away, although the specific uses of that money have not yet been determined. At the next council meeting, you will have to cast the deciding vote about which type of car to purchase in replacing the municipality's fleet.

	Option A (hybrid)	Option B (regular car)
Price (per vehicle)	$35,000	$22,500
Miles per gallon (MPG)	45	27
Amount of exhaust particles	Far below average	Average
Safety index	+++	+++
design	A+	A+

Which option will you choose: the hybrid vehicles (Option A) or the regular vehicles (Option B)?

- ❍ Option A
- ❍ Option B

Scenario 3: Cost2
Imagine you are about to design and build your next home. You have to decide what kind of heating system to install. You have a choice between two types of systems: a natural gas heating

system or a geothermal energy system. The natural gas heating system is worse for the environment since it makes use of a finite fossil combustible component – gas. Geothermal energy is clean, good for the environment, and infinite. However, installation costs for the geothermal system are approximately 30% higher than installation costs for the more traditional gas system. Which system would you choose: A natural gas heating system (System A) or a geothermal energy system (System B)?

- System A
- System B

Scenario 4: Cost2Table

Imagine you are about to design and build your next home. You have to decide what kind of heating system to install. You have a choice between two types of systems: a natural gas heating system or a geothermal energy system. The natural gas heating system is worse for the environment since it makes use of a finite fossil combustible component – gas. Geothermal energy is clean, good for the environment, and infinite. However, installation costs for the geothermal system are approximately 30% higher than installation costs for the more traditional gas system.

	System A (natural gas)	System B (geothermal)
Initial costs	low	high
Running costs	high	low
Energy output	high	high
Sustainability index	4 out of 10	9 out of 10

Which system would you choose: A natural gas heating system (System A) or a geothermal energy system (System B)?

- System A
- System B

Scenario 5: Social_NT

Imagine you need to purchase new furniture for your home. You are torn between a sofa made out of bamboo fabrics and a sofa made with more traditional fabrics. The bamboo sofa is a sustainable material and very environmentally friendly, however it only comes in somewhat outdated styles. The regular sofa is produced using bleaches, chemicals and synthetic fabrics, but comes in many modern styles. All other relevant factors (such as price, comfort and durability) are the same. Which option would you rather choose: The sofa made out of bamboo fabrics (option A) or the sofa made of traditional fabrics (option B)?

- Option A
- Option B

Scenario 6: SocialTabl
Imagine you need to purchase new furniture for your home. You are torn between a sofa made out of bamboo fabrics and a sofa made with more traditional fabrics. The bamboo sofa is a sustainable material and very environmentally friendly, however it only comes in somewhat outdated styles. The regular sofa is produced using bleaches, chemicals and synthetic fabrics, but comes in many modern styles. All other relevant factors (such as price, comfort and durability) are the same.

	Option 1 – bamboo	Option 2 - regular
Comfort	Comfortable	Comfortable
Style	Outdated	Modern
Price	$2,000	$2,000
Chemicals used in production	Few	Many
Sustainability	High	Low

Which option would you rather choose: The sofa made out of bamboo fabrics (option A) or the sofa made of traditional fabrics (option B)?
❍ Option 1
❍ Option 2

Scenario 7: Time
Imagine that you need to get your car washed and waxed, and received a free online coupon to do so. You can choose to get the regular wash & wax (option A), or you can choose the eco-friendly wash & wax (option B), which uses less water and uses cleaning products that are organic and do not harm the environment. When you arrive at the car wash, you learn that the regular wash takes 15 minutes, while the eco-friendly wash & wax takes 45 minutes. Which option would you choose: The regular wash (option A) or the eco-friendly wash (option B)?
❍ Option A
❍ Option B

Scenario 8: Building
Imagine you are the building manager of a large office block and you have to decide which heating policy to implement. You could regulate the maximum temperature of all heaters to not exceed a specified temperature. This would be good for the environment since it will regulate energy use and prevent waste. However, at times people might feel a bit cold and will have to put on an additional sweater. Alternatively, each heater could be regulated separately in each office and without temperature limits. In this case, people would always be cozy. However, a lot of energy might be used and potentially wasted (especially if people forget to turn their heat off at the end of the day). Which policy would you decide to implement: Temperature regulation (Option A) or unlimited self-regulation of temperature (Option B)?
❍ Option A
❍ Option B

Scenario 9: Kitchen
Imagine you are the CEO of a company and have to decide which policy to implement concerning the staff kitchen area. One option is to provide Styrofoam cups, which don't require a lot of effort for the employees since they can simply be thrown out. However, they are also bad for the environment because they never decompose. Another option is to implement a "bring your

own mug" policy, which would involve creating a rotating system of responsibility for filling the office dishwasher, letting it run at the end of the work day, and emptying it out the next day, which constitutes a significant amount of effort for your employees. However, this policy would be much better for the environment by reducing waste. Which option would you choose to implement: Providing Styrofoam cups (option A) or reusable mugs (option B)?

- ❍ Option A
- ❍ Option B

Scenario 10: Benefits

Imagine that you are the CEO of a small start-up company that is deciding whether to enact environmentally friendly practices. These include installing eco-friendly lighting fixtures and appliances, and using more expensive, organic office cleaning supplies. However, putting these measures in place would be quite costly for your small company, and would mean workers would have to forgo the free lunch you were planning to provide every Friday. Which option would you choose: Putting environmentally friendly practices into place (option A) or offering free Friday lunches (option B)?

- ❍ Option A
- ❍ Option B
